# Influencing Factors In-Hospital School Education: Exploring the Context From the Teacher’s Perspective

**DOI:** 10.5334/cie.126

**Published:** 2025-01-31

**Authors:** Francisca Jiliberto, Nair Zárate Alva

**Affiliations:** 1 University of Barcelona, Spain; 2 Universitat Autònoma de Barcelona, Spain

**Keywords:** Hospital school, hospital teacher, hospitalised student, teaching

## Abstract

Hospital teachers face unique challenges and require specific skills to cope with the demands of teaching within a hospital setting effectively. Additionally, the quality of education in hospital schools (HS) may be affected by factors such as resource availability and coordination with other professionals. This initial study examined the factors influencing education in HSs for hospitalised children with physical health conditions. Teachers from the eight HSs in Catalonia (*N* = 16) responded to an online questionnaire developed based on a scoping literature review that identified factors reported to influence education in HSs. Employing a mixed-method convergent parallel design, quantitative data from closed-ended questions and qualitative data from comments were analysed in parallel. Results showed that hospital teachers face challenges such as adapting to diverse educational stages and subjects, selecting teaching methods tailored to students’ individual needs, and coordinating with various professionals on a case-by-case basis. They often lack sufficient budgets, consistent access to resources, and opportunities for professional training. Participants’ views on the skills required by hospital teachers align closely with literature findings, as does their perception of the emotional impact of working in a HS. Understanding the unique context of HSs is crucial for stakeholders and policymakers to ensure inclusive and equitable quality education for all.

Hospital schools (HS) are indispensable to “ensure inclusive and equitable quality education and promote lifelong learning opportunities for all,” as defined in the 2030 Agenda (UNESCO, 2016). Notably, about 20% of school-age children live with a significant medical condition that could lead to school absences, including hospitalization, a number that is likely to increase due to medical advances (Compas et al., 2012; Nasir et al., 2019).

During periods of hospitalisation, it is the hospital teacher who assumes educational responsibility. The following sections will explore what is currently known regarding the unique educational context of hospital schools, the hospital school teacher’s role, the qualities required for hospital teachers to cope effectively, and the emotional impact of working in a hospital school.

## The Unique Educational Context of Hospital Schools

Over the past decades, the focus of hospital care has shifted from being disease-centred to being person-centred. That is, the current focus is on the patient and their family environment in an effort to promote capabilities and possibilities (Molina et al., 2019). Nevertheless, hospitalisation drastically distances students from their usual educational environment (Alguacil et al., 2015) and can provoke multiple, often stressful, reactions involving both the child and their family (Muñoz-Violant et al., 2023).

Given this reality, HSs should ensure the “provision of teaching and learning away from school; focus on student-centeredness; strengthening home-school-hospital partnerships; facilitation of teacher and peer acceptance; school re-entry support; resources and funding; and advocacy” (Plage et al., 2022, p. 1). As such, they include essential aspects of educational continuity such as helping to normalise the illness situation (Hen & Gilan-Shochat, 2022; Kontogianni et al., 2021), providing a purpose, and enabling the student to maintain their academic and social life (Andreatta et al., 2016; Choquette et al., 2016). Specifically, according to Capurso and Dennis (2017), six key educational factors should be considered: “relationships, making sense and constructing knowledge, assuming roles in front of others, metacognition, individualities and inter-institutional communication” (p. 158).

In addition to the unique demands placed on teachers, a lack of time, support, and recognition, along with the absence of opportunities for continuous training are characteristics of many in-hospital schools. Finally, allocation of economic resources is an important factor in HSs (Işiktekiner & Altun, 2011) besides, a lack of standardisation among HSs in terms of structure and organisation (Álvarez, 2013; Andreatta et al., 2016; Steinke et al., 2016).

## The Hospital School Teacher’s Role

HSs cater to students of different educational levels coming from different educational centres of origin that, in turn may apply different methodologies (Carstens, 2008; Keehan, 2019). As a result, the hospital teacher needs to collect individual information from each student while coordinating their actions with hospital professionals, professionals from the educational centres of origin, and other educational support teams (Dixon, 2014). In addition, it is also the responsibility of the hospital teacher to find a sensitive and respectful approach to the situation that the student is experiencing (Keehan, 2021; Latorre & Blanco, 2010) while designing educational programs and interventions appropriate to the student’s physical and emotional condition. That is, the hospital teacher must promote a sense of normality, routines, and motivation to learn while maintaining communication with the student’s regular school for academic goals and social aspects, such as fostering communication with peers (Boles & Winsor, 2019).

Furthermore, hospital teaching involves dealing with uncertainty on a daily basis in terms of number of students, days of care, duration of educational program, and other circumstances that are usually rather predictable in regular schools (Andreatta et al., 2016). Similarly, considerably more time is spent communicating with both students and families in the HS (Steinke et al., 2016). Therefore, hospital teachers must find a balance in allocating time for curriculum development, communication with students, and fostering contact with their peers and the educational context of origin (Carstens, 2008; Keehan, 2019).

Finally, assessing how much effort can be asked of students given their health condition may require constant evaluation. On a more positive note, these individualised, learner-centred approaches often result in the establishing a very close teacher-learner relationship, which is not common, or possible, in regular schools (Carstens, 2008).

As summarised by Andreatta et al. (2016), hospital teachers must constantly keep in mind and adapt to the four variables of space, time, students, and activities. The quality of education in HSs is determined by the management of unforeseen events, the appropriateness of educational interventions and programs, and the class climate established.

## Attributes Required for Hospital Teachers to Cope Effectively

Several authors describe the attributes required for hospital teachers to cope effectively within a hospital setting:

- “Passion, sympathy, empathy, flexibility, teamwork and organisational skills” (Carstens, 2008, p. 56)
- “Being patient, loving, happy, open to communication, positive, emotionally stable and sensitive to the disease situation and special needs” (Işiktekiner & Altun, 2011, p. 2)
- Being communicative, creative, collaborative, cooperative and resilient (Alguacil et al., 2015)
- “High motivation, ability to care, ability to overcome obstacles, dedication, creativity and perseverance” (Steinke et al., 2016, p. 40)

Sukhanova and Sharikov (2020) categorized hospital teacher attributes in the following three areas: professional knowledge and competencies, personal qualities and characteristics, and behavioural norms and values. Furthermore, they considered the following additional requirements and standards: hygiene and infection control protocols, medical knowledge and sequelae, laws and regulations governing hospital pedagogy, and skills for teaching in an unconventional educational setting.

## The Emotional Impact of Working in a Hospital School

Hospital teachers may face circumstances that may take a great emotional toll on them (Hen, 2020), such as:

- life-threatening events (Keehan, 2019),
- abrupt changing health prognoses (Carstens, 2008),
- situations of daily suffering (Steinke et al., 2016), and
- families experiencing stress and confusion, possibly fragmented if relocated to be near the hospital, and with their usual routines disrupted (Benito et al., 2011).

Capurso and Dennis (2017) highlighted the characteristics of a hospital and the events that occur in it as a set of situations that can emotionally alter both the learner and the hospital teacher. The challenge of the hospital teacher is to counteract these effects, at least while delivering educational care.

Further, Bustos and Cornejo (2014) mentioned other challenges such as reaction to the death of a student, the transition to palliative care and the bond with the family. Carstens (2008) added that “the aspects that most concern hospital educators are staying emotionally healthy and coping with losses” (p. 38).

All these experiences can affect hospital teachers emotionally. Therefore, it is highly recommended that hospital teachers receive training in defusing (Benito et al., 2011; Lizasoáin & Lieutenant, 2002), maintaining limits, adapting to work with a reduced team or without one (Lemke, 2004), and dealing with few opportunities for collaboration (Steinke et al., 2016).

Despite these challenges, Keehan (2019) noted that hospital teachers find the following aspects satisfying:

- the educational experience itself,
- supporting students during challenging times,
- providing moments of socialisation, normalcy, and familiarity where students’ minds move away from the illness situation, and
- witnessing students’ recovery and discharge.

Finally, being a hospital teacher is often considered a source of professional growth (Carstens, 2008) and highly satisfying (Hen & Gilan-Shochat, 2022; Steinke et al., 2016).

## The Present Study

The present study aimed to answer the following research question: What factors influence education in HSs in Catalonia, Spain, as perceived by hospital teachers? By addressing this question, the goal was to provide valuable insights for educators and policymakers in developing strategies to optimise education in HSs.

In Catalonia, hospital students treated for mental health conditions receive educational care through separate educational units not considered to be part of HS while following distinct educational, procedural, and management approaches. Therefore, the students of participating HS teachers suffered from physical health problems. Another characteristic of the Catalan setting is that compulsory education goes up to the age of approximately 16 years old, or what would be tenth grade in the American system. That is, while 11th and 12th grades are compulsory in America, their Catalan equivalent (“batxillerat”) is not. Therefore, education in Catalonia is organised into four stages: infant (3–5 y/o), primary (6–11 y/o), secondary (12–15 y/o), and post-compulsory education (over 16 y/o).

At the time of the study, there were eight HSs in Catalonia, with a total of 16 hospital teachers working in them. Half of all HSs were comprised of a single teacher; the largest HS had five (see Table 1).

**Table 1:** Participating Hospitals, Their Healthcare Level, Teachers, and Location.


HOSPITAL	HEALTHCARE LEVEL	NUMBER OF TEACHERS	LOCATION (CITY)

Hospital Vall d’Hebron	3	5	Barcelona

Hospital Sant Joan de Déu	3	3	Barcelona

Hospital de la Santa Creu i Sant Pau	3	1	Barcelona

Hospital Dr. Josep Trueta	2	1	Girona

Hospital Germans Trias i Pujol	2	1	Badalona

Parc Taulí – Hospital Universitari	2	2	Sabadell

Hospital Arnau de Vilanova	2	1	Lleida

Hospital Sant Joan de Déu	2	2	Manresa


*Note*. Healthcare levels refer to the available medical technology and equipment. Third-level care hospitals handle high-complexity cases from all over Catalonia, as secondary-level hospitals do not offer specialized services.

Finally, hospitals in Barcelona (third-level care hospitals) take in high-complexity cases from all of Catalonia, as hospitals outside the city (second-level hospitals) do not offer specialized services with more advanced technology and sophisticated medical equipment. Therefore, hospital teachers working in Barcelona are likely to attend to students and families who have had to relocate during hospitalisation periods and, therefore, are even farther removed from their original environment.

## Method

### Participants

All hospital teachers working in Catalonia and not involved in the study as researchers (*N* = 15) were invited to participate. The principal investigator of the study was a hospital teacher in Catalonia but was excluded due to their involvement as an author.

Thirteen hospital teachers agreed to participate, representing 87% of the target population (or 81% of all active hospital teachers in Catalonia, if we include the principal investigator). At the time of the study, the entire target population were women. Due to the small size of the target population, the socio-demographic data gathered were limited to ensure anonymity. All participants were over 30, and more than half were older than 50 years old.

Six participants (46.2%) worked in HSs located in the city of Barcelona, and seven (53.8%) worked elsewhere in Catalonia. Teachers working in mental health units were not part of the target population since, as mentioned, care for students with mental health conditions in Catalonia is provided through separate educational units outside of HSs while following specific educational, procedural, and management approaches different from those of HSs. Participants did not receive any compensation or incentives for their participation.

### Design

A mixed-method convergent parallel design (Creswell & Plano, 2018) was employed. A scoping literature review was first carried out to identify factors previously described to influence education in HSs. Based on these findings, an online questionnaire was developed, including close-ended questions and a comments section for each question. Quantitative data from close-end questions and qualitative data from written comments were collected and analysed in parallel while merging the results for an overall interpretation.

As described by Creswell and Plano (2018), this design is suitable when researchers aim to compare and contrast quantitative results with qualitative findings for corroboration and validation purposes. Furthermore, the combination of quantitative and qualitative methods allowed for the design of confirmatory and exploratory questions simultaneously (Teddlie & Tashakkori, 2012). While close-ended questions probed findings from previous studies, the comments section was added to further explore the participants’ perspectives. When applying this design, qualitative items do not result in a complete context-based qualitative data set. Instead, they can provide additional themes and interesting quotes that may validate and embellish the quantitative survey findings (Creswell & Plano, 2018).

At the time of the study and to the authors’ best knowledge, no survey for hospital teachers inquiring about factors that may influence education in HSs had been developed. Therefore, creating an instrument that could quickly capture the working conditions of hospital teachers and their perspective on factors that may influence education in HSs was an attractive pursuit. This pragmatic approach favoured the feasibility of an online survey over more time-consuming alternatives, such as in-depth interviews, to better adapt to the hospital teachers’ complex context and limited availability.

### Instruments

The development and validation of the questionnaire constituted a fundamental aspect of this research. A scoping literature review (Armstrong et al., 2011) was performed to identify factors previously found to influence education in HS and serve as a basis for developing the questionnaire. Research articles and reviews in English and Spanish and up to 10 years old were retrieved in November 2021 from Google Scholar, Dialnet, Scopus, REDINED and ERIC bibliographic databases using the search terms “hospital school,” “hospital teacher,” “hospitalised students,” and synonyms. Articles were included through a multi-phase selection process (see Supplementary File 1).

Factors found to influence education in HS extracted from the literature review were grouped into three categories: pedagogical, socioemotional, and hospital context-related (see Table 2).

**Table 2:** Pedagogical, Socioemotional, and Hospital-Context-Related Factors That May Influence Education in Hospital Schools.


PEDAGOGICAL FACTORS	SOCIOEMOTIONAL FACTORS		HOSPITAL CONTEXT-RELATED FACTORS

	COMPETENCY PROFILE	EMOTIONAL IMPACT	

Educational stage Subject specialisation Teaching methodology Lesson adaptations Materials and resources Class climate Lesson duration Beginning of coordination with school of origin Means of communication with school of origin Contact periodicity with school of origin Programming Coordination with other professionals Financing Workplace Technological resources Specific professional training Supervision and support Value given to being a hospital teacher	Flexibility Serenity Creativity Dedication Perseverance Patience Happiness Empathy Positivity Communication skills Fostering positive personal relations Emotional stability Kindness Openness to communication Sensitivity towards the illness situation Harmony with oneself Passion for life, students and work Ability to inspire hope and prospects for the future Ability to create a positive classroom environment Acceptance towards students’ and family’s emotions Stress-reducing capacity	Deterioration of health conditions Transition to palliative care End-of-life process Need for debriefing Seeking support Satisfaction in regard to being a hospital teacher Positive professional experience Teaching as a normalising tool Admiration for the students’ trust and dedication Satisfaction when providing support Valuing the relationships established with the pupils Joy when reducing anxiety, stress and suffering Joy upon students’ recovery and discharge Satisfaction regarding family responses	Sense of belonging Specific hospital training Daily information transfers Professional needs Working space Means of communication Availability of classrooms Use of personal material resources Access to daily student information Interruptions Hospital school accessibility Uniform availability Uniform distinction


*Note*. The above factors were extracted from the literature review.

*Pedagogical factors* encompassed those directly related to teaching, such as lesson times, teaching methodologies, or coordination with education professionals in mainstream schools. *Socioemotional factors* were divided into a competency profile section, consisting of 21 attributes previously described as required for hospital teachers to cope effectively with circumstances within a hospital setting, and an emotional impact section, consisting of 14 items related to the emotional impact of working as a hospital teacher extracted from the literature review. *Hospital context-related factors*, in turn, included hospital characteristics that could influence the hospital teacher’s work, such as the transfer of information and coordination within the hospital or the need for specific hospital training.

Multiple-choice questions were used for pedagogical and hospital context-related factors. Some of these questions included non-mutually exclusive answers (e.g., in which educational stage/s do you teach?), allowing participants to select more than one answer. Conversely, 5-point Likert scale questions were employed for the socio-emotional factors. For the competency profile, participants were asked to rate each attribute in terms of them being “very,” “fairly,” “moderately,” “slightly,” or “not” necessary for the hospital teacher. For the emotional impact section, each item extracted from the literature review was translated into a first-person statement (see Supplementary File 2). Participants were asked to rate their agreement with each statement, selecting “totally agree,” “quite agree,” “indifferent,” “quite disagree,” or “totally disagree.” Since some statements referred to situations that not all participants may have experienced, the option “not applicable” was also included for this question.

This first version of the questionnaire was subjected to validation by four experts:

- Professor of Special Education and Hospital Pedagogy with more than 30 years of experience. Vice-Dean of the Education and Psychology Faculty at their university.
- National coordinator for hospital schools and homebound educational care at the Ministry of Education with more than 20 years of experience.
- Coordinator for an NGO supporting hospital schools with more than 20 years of experience.
- Hospital teacher with over 10 years of experience in HSs.

Based on their feedback, a revised updated questionnaire was then subjected to validation by two hospital teachers from outside Catalonia (see Supplementary File 3 for a summary of the expert validation process). The questionnaire was deemed validated with 70 items in total:

- Four items for socio-demographic data
- 18 items for pedagogical factors
- 35 items for socio-emotional factors
  - 21 items concerning the competence profile
  - 14 items concerning the emotional impact of working in a HS
- 13 items for hospital context-related factors

Participants were encouraged to provide comments by the following prompt: “You will find a space for comments in each of the questions; remember that this may enrich and clarify the answers given.” Further, following expert advice, participants were allowed to leave questions unanswered (see Supplementary File 4 for a description of the six items that received partial responses). An unofficial translation of the survey questions may be found in the supplementary materials (Supplementary File 5), and the original survey is available upon request from the corresponding author.

### Procedure

All members of the target population were invited to participate via their education department institutional email accounts (accessed through the principal investigator), with a participant information sheet attached, in March 2021. Upon acceptance of the invitation, an online questionnaire allowing a response period of 10 days was sent to each participant.

### Data Analysis

Quantitative data from the close-end questions were gathered using an Excel template and an SPSS data matrix. This required prior numerical coding of each item, data entry, transformation of syntactic data into numerical data, value labelling, and data cleaning.

The small sample size prevented estimation of correlation coefficients and restricted the potential for inferential analysis (Delgado, 2014). To achieve a confidence level of 95% for a population of 15 people, 100% of the population would have had to participate. For this reason, only descriptive statistics were employed.

Qualitative data from a total of 98 comments, comprised of, 2330 words, were analysed following thematic content analysis (Hernández-Sampieri & Mendoza, 2018). This analysis aimed to capture any additional themes from the comments. At the same time, illustrative quotes were selected to be presented along with the quantitative results.

Quantitative and qualitative analyses were conducted in parallel, and upon completion, results were combined for an overall interpretation. FJ conducted the analyses, and NZ validated them.

### Consent

Participants were electronically presented with an informed consent form to be filed before the completion of the questionnaire. Completion of the questionnaire was anonymous, and no user data, including email, were stored.

## Results

This study aimed to examine the factors that influence education in HS for hospitalised children with physical health conditions in Catalonia, Spain, to guide educators and policymakers in developing strategies to optimise education in HS. Factors previously described as influencing education in HS were identified through a scoping literature review and were subsequently used to develop a survey for hospital teachers that also included a comments section encouraging them to voice their perspectives.

Thirteen hospital teachers completed the survey, representing 87% of the target population. All participants were women over 30 years old, and over half were older than 50 years old. Around half of the sample worked in hospitals in Barcelona. More than half of the participants reported having more than five years of experience teaching in HS. The entire sample had more than five years of teaching experience in mainstream schools, while 77% had over 11 years of experience in this setting.

### Pedagogical Factors Influencing Education in HS

#### Hospital Teachers Must Adapt to Working Across Multiple Educational Stages and Subjects

As mentioned, education in Catalonia is organised into four stages: infant (3–5 y/o), primary (6–11 y/o), secondary (12–15 y/o), and post-compulsory education (over 16 y/o). In most cases, mainstream teachers work either in primary or secondary education. Secondary education teachers specialise in one subject and may also teach in post-compulsory stages, as most mainstream schools offer two years of post-compulsory education leading to university or professional degrees.

Results revealed that this pattern is not followed in HS, however. The entire sample reported teaching both in primary and secondary education, while 38% reported working with students from all four educational stages. Moreover, 62% reported to teach any subject required.

Qualitative data revealed that teachers specialised in primary stages might encounter difficulties while working with students from higher levels.

“At the post-obligatory level […] is where I have more difficulties, since I am a primary school teacher…” (Participant 3)

However, comments highlighted how, in these cases, the role of the hospital teacher may not be focused on teaching per se.

“The care of students in post-compulsory stages is more focused on guidance, document management, coordination with centres [educational centres], or any other necessary procedures, rather than direct attention to curricular aspects.” (Participant 9)

#### Teaching Methodologies, Adaptations and Materials Are Selected Based on Students’ Needs

The entire sample described combining different methodologies depending on the situation to facilitate student comprehension. Similarly, all participants reported making adaptations, whether procedural, content-related, or otherwise as required by the students’ health conditions. These included adjusting working postures and/or imparting class in treatment spaces or during simple medical procedures. Likewise, there was agreement upon the need to select materials and resources to promote learning based on students’ characteristics.

“In short-term admissions, I use more educational materials or games adapted to the student’s age. In longer admissions, I alternate activities that require paper and pencil and materials/games. It also depends on the student’s motivation: Some students ask to follow curricular content and work more with paper and pencil […] [while others] need more time with games…” (Participant 9)

Most respondents (85%) reported creating daily individual plans for each student. In the case of long-term hospitalisations, over half of the participants (54%) reported also using weekly plans. However, qualitative data showed that these plans may vary to adapt to students’ needs.

“There are times when, due to the student’s particular situation in the hospital, we must change everything planned and re-schedule on a daily basis. For instance, when they’re not feeling well because of treatment, and we, therefore, cannot hold classes every day, or when their health worsens… you often need to be flexible in this regard.” (Participant 4)

All respondents considered class climate to be important. Indeed, 96% regarded it as an indispensable aspect to consider while aiming to create a relaxed and motivating class climate where the student can feel comfortable and calm.

“I try to make the lesson a positive experience for the student, not just another stressor in the hospital setting.” (Participant 9)

While most mainstream schools follow defined class times and schedules, survey responses showed variability in this regard for HS. Over half of the participants provided comments on this item. Qualitative data revealed that the duration of classes varied and depended on several factors related to the hospital context.

More than half of the participants (61%) reported having access to a classroom with all the necessary materials for teaching. However, most participants (92%) reported delivering instruction in the students’ hospital room, while some (38%) reported imparting classes in treatment areas.

#### Hospital Teachers Coordinate With Education, Psychosocial, and Health Professionals as Needed Case-by-Case

According to education regulations in Catalonia, the mainstream school that the pupil typically attends must provide an individualised work plan for the hospitalised student. Furthermore, grades that go into academic reports are always given by the mainstream school and not the HS. For this reason, coordination among the two settings is vital.

Most participants (69%) described initiating contact with the student’s mainstream schools on the first day after meeting the student. Some participants (31%) reported only initiating contact if the anticipated duration of the hospitalisation was more than five days, while others (23%) did so only for hospital stays over 10 days. Most participants described using phone calls (80%) and/or email (85%) to communicate with mainstream schools; some (23%) also used video calls.

After first establishing contact with the mainstream school, the frequency of communications varied greatly among participants. Further, this item elicited the most written comments, as all participants but one (92%) commented on it.

“The frequency is very variable depending on the age of the pupils and the predisposition of the centre. An attempt is made to follow up with the centre according to needs.” (Participant 2)

“There are times when it is only necessary to do it once, at the beginning of the admission; at other times, it is necessary to do it every day.” (Participant 1)

**Figure 1:**
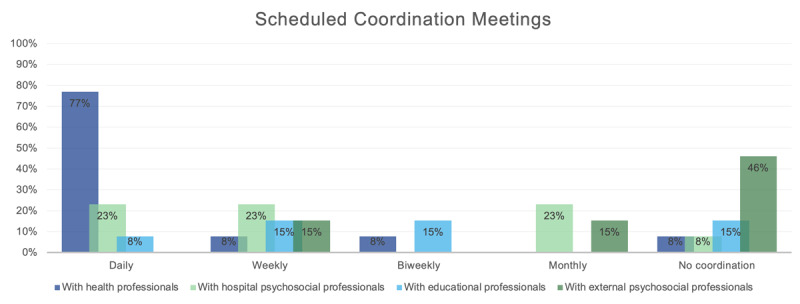
Frequency of HS Teachers’ Regularly Scheduled Meetings With Other Professionals.

In addition to coordinating with mainstream schools, hospital teachers may also need to coordinate with health professionals and psychosocial professionals, such as psychologists and social workers (either from the admitting hospital or external). When asked about regularly scheduled meetings for such coordination, 77% of participants reported having daily meetings with health professionals, while the frequency of meetings with other professionals varied greatly (see Figure 1).

#### Hospital Schools Lack Annual Budgets to Meet Their Needs, and Access to Resources Varies

Mainstream public schools in Catalonia receive yearly budgets from the regional government. This is not the case for HS, as they depend upon hospitals for this matter. As a result, most participating hospital teachers (69%) reported that they did not have a previously defined budget to meet their needs, but hospitals may provide materials upon request. Some participants (23%) report having a budget dependent on previous approval.

In terms of technological resources, almost all participants (92%) reported having access to a desktop computer with internet, a desktop phone, a photocopier, and a scanner. Nevertheless, around half of them reported not having access to a mobile phone for communication within the hospital or a personal laptop. All respondents reported using personal materials when not available in their workspace.

#### Most Hospital Teachers Do Not Have Access to Specific Professional Training, and Some Lack Sufficient Supervision and Support

The Catalan Education Department offers free and recognised professional training for teachers on a variety of topics. However, results showed that 67% of the hospital teachers in this study reported not having access to professional training relevant to teaching students with a medical condition. Twenty-five percent reported having access to specific professional training outside of the offerings of the Education Department.

Among respondents, 69% reported having supervision and support. However, of these, 22% described such services as not quickly accessible and efficient. Some hospital teachers (15%) described not having sufficient supervision and support, and some (15%) abstained from responding.

In terms of how professional development was valued, 54% described their professional experience as hospital teachers to be a positive professional challenge, and 77% reported it to be a source of professional growth. All participants but one (92%) also described it as a source of personal growth. No participants agreed with the description of their professional experience as being exhausting.

**Figure 2:**
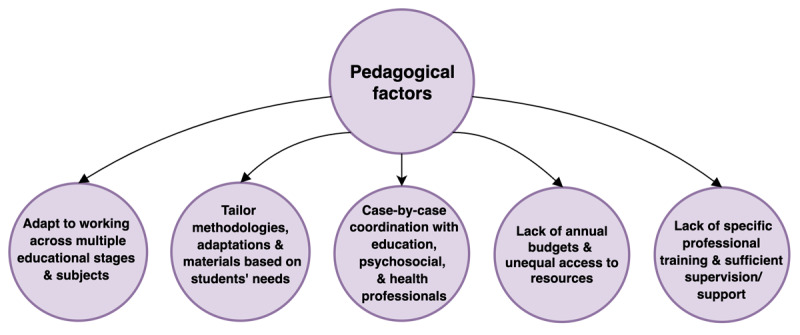
Pedagogical Factors Influencing Education in HS.

A summary of findings from this section is provided in Figure 2.

### Socioemotional Factors Influencing Education in HS

#### Participants’ Views on the Attributes Required by Hospital Teachers Align Closely With Literature Findings

A 5-point Likert scale question probed 21 attributes described in the literature as being required by hospital teachers to cope effectively with circumstances within a hospital setting.

**Figure 3:**
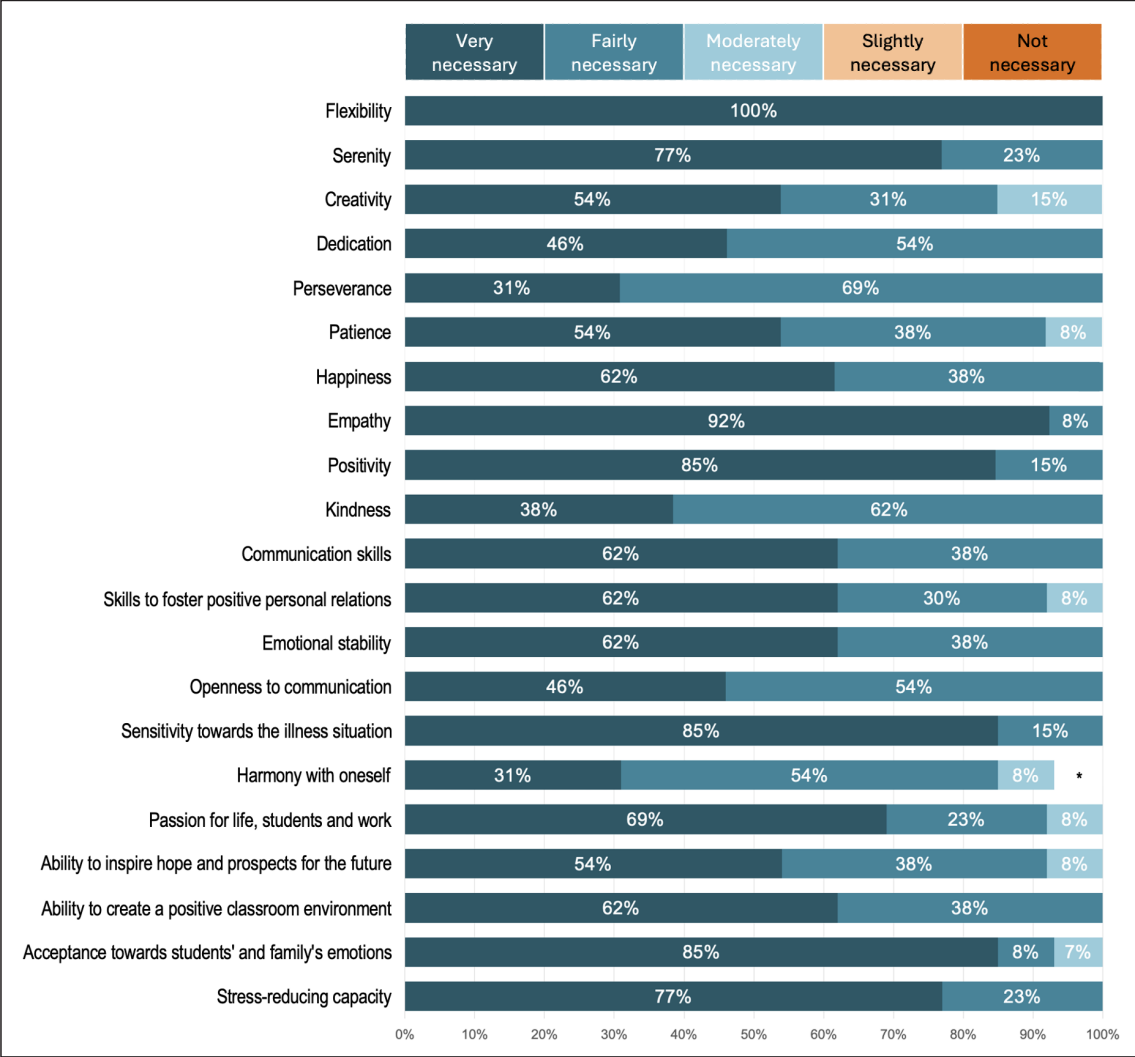
Competency Profile of Hospital Teachers. *One participant did not respond to this item.

The responses of the participants were robustly aligned with evidence from the literature review (see Figure 3). Fourteen attributes were unanimously described as “fairly necessary” or “very necessary,” with none labelled as “not necessary” or “slightly necessary.” Flexibility and empathy were described as “very necessary” attributes by 100% and 92% of participants, respectively. The following attributes were also checked as “very necessary” by more than 75% of participants: “positivity,” “serenity,” “stress-reducing capacity,” “sensitivity towards the illness situation,” and “acceptance towards students’ and family’s emotions.”

#### Statements From the Literature About the Emotional Impact of Being a Hospital Teacher Obtained Widespread Agreement From Participants

**Figure 4:**
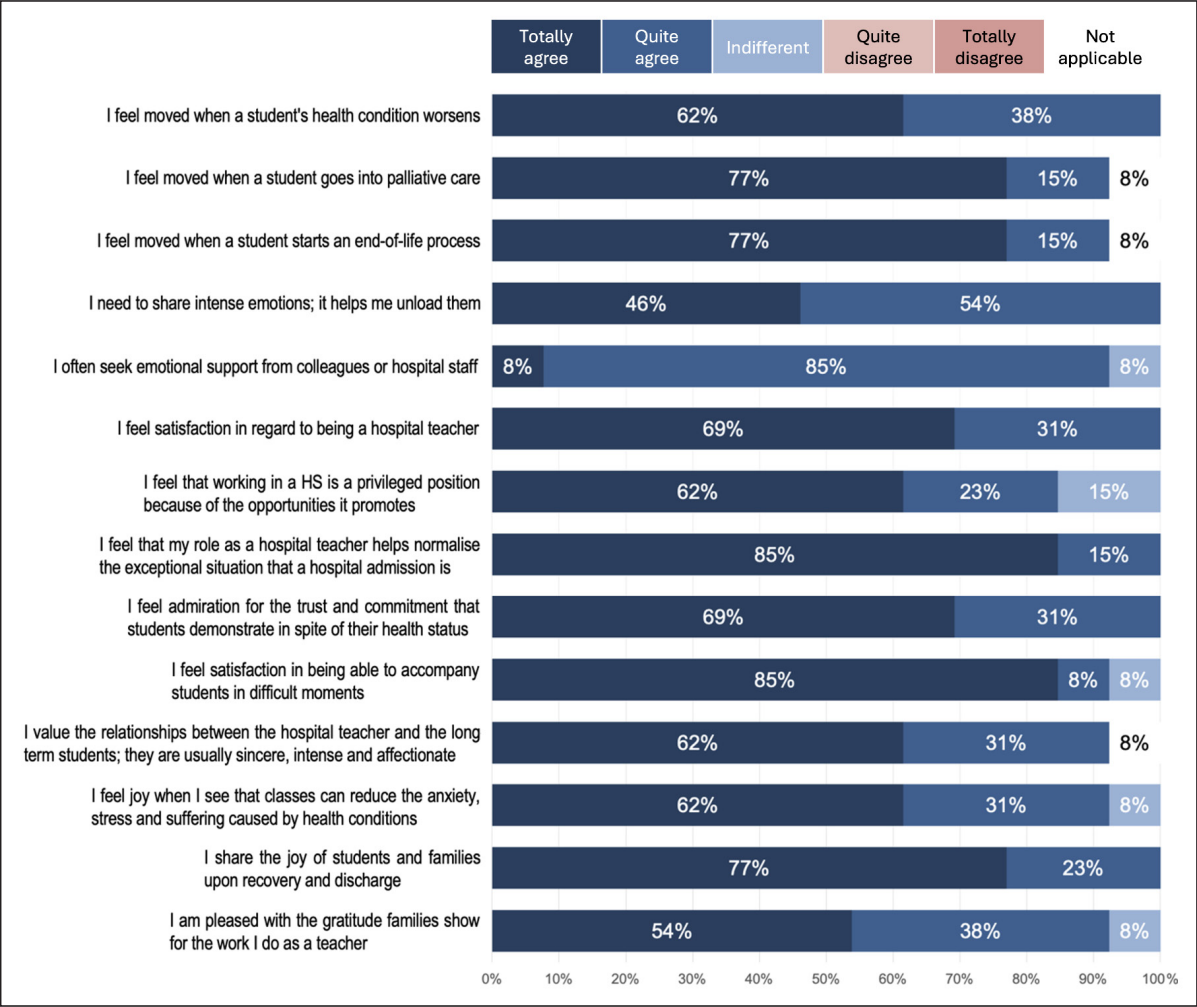
Emotional Impact of Working as a Hospital Teacher.

Another Likert scale question asked participants to rate their agreement towards 14 statements about the emotional impact of working as a hospital teacher extracted from the literature review (see Figure 4). For this question, participants were also allowed to select “not applicable” (as they may have not been exposed to the situation described in the statement).

Once again, our findings showed strong agreement with the literature. No participants disagreed with any of the presented statements, and all items except one received ratings of “quite agreed” or “totally agreed” from more than 90% of participants. While the first five statements referred to situations that could be described as challenging, the remainder described positive feelings or emotions. However, no distinct pattern was observed among answers for these categories. Participants tended to agree with both challenging and positive experiences described in the statements.

The statements with the highest scores for “totally agree” were:

- “I feel satisfaction in being able to accompany students in difficult moments” (85%),
- “I feel that my role as a hospital teacher helps normalise the exceptional situation that a hospital admission is” (85%),
- “I share the joy of students and families upon recovery and discharge” (77%),
- “I feel moved when a student goes into palliative care” (77%),
- “I feel moved when a student starts an end-of-life process” (77%).

### Hospital Context-Related Factors Influencing Education in HS

#### Hygiene and Safety Training and Access to Student Medical Information Varies Across HS

Hospital teachers require hygiene and safety training in adherence to infection control protocols. However, in the current study only 23% reported having received such training upon taking up their position; the rest reported having learned through experience and by imitation. Thirty-one percent described having acquired this knowledge from another hospital teacher.

The student population of HS may vary daily depending on admissions. Hospital teachers may access information about their caseload by checking the hospital census or being informed by healthcare professionals. Results showed that 69% of hospital teachers consult the hospital census, while 46% also report being informed by nurses.

As the students’ health status may vary daily, having updated access to students’ medical information is useful for hospital teachers so they can tailor their approach towards students and their families. However, 38% respondents reported only having access to this information when they requested it. One participant described not having access to students’ medical information beyond what was shared by the families or students themselves.

“I ask the nurses as we cross paths on the hallways. When I have to enter a new student’s room, if there has been a change in isolation, I need information about whether they can leave the room…” (Participant 9)

### Despite Their Distinctive Uniforms, Hospital Teachers Are Not Always Easily Identifiable, Nor May the Hospital School Be Easily Found

In a hospital context, being able to identify hospital teachers and/or locating the HS is useful for students and families. Nevertheless, 62% of participants reported that the HS was not easily found. Ninety-two percent of hospital teachers reported having a uniform provided and maintained by the healthcare centre, while one participant stated otherwise. Almost half (46%) of the sample considered their uniform to be easy to recognise and indicative of their role, as distinct from healthcare personnel.

#### All Hospital Teachers Report Having Interruptions During Instruction

All hospital teachers must adapt to having interruptions while teaching. Fifty-four percent reported having many interruptions during classes.

“Students are continually undergoing tests, analyses, paediatric visits, and they are even waiting to go down to the operating room. As a result, the educational session can be interrupted at any time for extremely more relevant reasons. Most of the time, once the tests, visits, etc., are finished, the sessions are resumed.” (Participant 1)

One participant pointed out that classes could serve as a distractor during some medical procedures. Moreover, they described how having medication delivered in the classroom in the company of peers could promote conversations around these experiences.

“Nurses often supply medications in the classroom while we are working. I believe that these interruptions are not bad because they help students interact with each other and talk about issues that are important to them. With nurses it took some time, but now they value it as very positive to deliver the medications in the classroom while the child is alone [without the family present] because it is easier for them and the child is more cooperative and less anxious.” (Participant 11)

#### Not All Hospital Teachers Feel a Sense of Belonging Towards the Hospital

Eighty-five percent of hospital teachers perceived that the hospital paid attention to their professional needs. Of these, 55% reported that solutions were not always offered to meet their needs. On the contrary, 15% responded that their needs were only occasionally or rarely heard and that solutions were never offered. When asked about their sense of belonging towards the hospital, 69% reported feeling included and valued, while 15% expressed not feeling this way.

**Figure 5:**
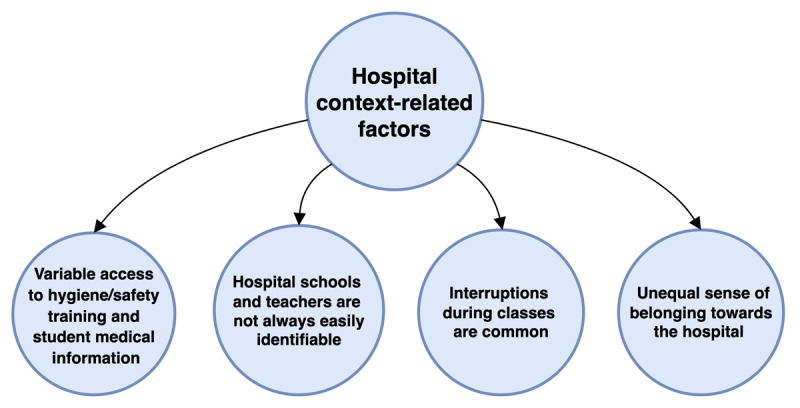
*Hospital Context-Related Factors Influencing Education in HS*.

A summary of findings from this section is provided in Figure 5.

### Additional Themes From the Qualitative Data

The findings from the qualitative data were presented previously alongside the quantitative results. However, the thematic analysis revealed an additional theme: lack of coordination between hospital schools.

“I think it is very important, fundamental, I would say, to be able to share our work with at least one colleague. Not to be alone in educational care, so as not to feel alone.” (Participant 3)

“I think it is important to mention the lack of closer coordination between hospital schools, to share ways of working, ideas, or to make a more detailed transfer.” (Participant 4)

The items that received the most comments are quantified below, as this may highlight those topics that participants felt more compelled to express their perspectives on:

– Frequency of communication with the mainstream school: 12 comments
– Class interruptions: 8 comments
– Coordination with other professionals: 7 comments
– Duration of lessons: 7 comments
– Access to student information: 6 comments
– Initiation of coordination with the school of origin: 5 comments
– Coordination with the school of origin: 5 comments

## Discussion

The present study examined the factors that influence education in HS for hospitalised children with physical health conditions in Catalonia, Spain. As shown in above, this is a unique context, different from regular schools in many aspects (Benigno & Fante, 2020; Steinke et al., 2016). As mentioned by Muñoz-Violant et al. (2023), the situations experienced by students and families in a hospital are often stressful and are marked by uncertainty, lack of privacy and being uprooted from one’s usual environment, among others. Furthermore, at another level, the priorities in a hospital environment are the patient’s health status, its control, the procedures that need to be applied and other activities that are not found in a regular educational context.

### Pedagogical Factors

Generally, teachers teach at a specific educational level and specialise in a specific subject. However, the entire sample in this study reported teaching both primary and secondary education, and 62% reported teaching any subject required. Altogether, it can be inferred from our findings that hospital teachers cater to a wide diversity of pupils, varying in age, stage, level, subject, learning style, and adaptations specific to a given disease situation, which coincides with the findings of Sukhanova and Sharikov (2020), Keehan (2019), and Äärelä et al. (2018). Congruently, 100% of participants described that the adaptation and methodologies used varied according to the student and the needs of the moment, suggesting the need for a high capacity for flexibility, which coincides with Benigno and Fante (2020), Capurso and Dennis (2017), Steinke et al. (2016), Burns (2013), and Carstens (2008). Furthermore, our qualitative data underscore how instructional plans are constantly adapted based on students’ needs. Ninety-six percent of the sample regarded class climate as an indispensable aspect to consider, the goal being an environment where the student feels comfortable, calm, relaxed and motivated, which coincides with Bustos and Cornejo (2014).

As Steinke et al. (2016) observed, the duration of classes often varies depending on the student, the day, and the medical tests to be performed, among others. Similarly, both our quantitative and qualitative results described a wide variability concerning the duration of classes, determined by the circumstances of the moment. Moreover, all participants reported experiencing interruptions during classes, which Benigno and Fante (2020) also mentioned as frequent in the hospital context.

The challenge of educating such a diverse student body makes it essential for hospital teachers to be restless professionals in search of new knowledge and skills, innovative methodologies, and creative use of resources, not to mention the need to be engaged in constant discovery of the latest information and communication technology, including the many resources offered online. Undoubtedly, these professionals must go beyond the teaching category to which they are assigned to look for solutions to expand their capacities and skills as teachers.

Ideally, the students feel a sense of belonging to the educational centre of origin, which the hospital teacher tries to maintain and promote. For this reason, it is important to direct efforts towards what Pini et al. (2019) referred to as the “interconnectedness of health, educational, developmental, and social aspects” of students in situations of illness. However, our results showed that the frequency of communication with regular schools varied greatly. Interesting, this item was the most commented (by 92% of participants). Qualitative findings stressed how the frequency of communications varied case-by-case.

A similar pattern was observed for instructional student plans; that is, they are designed in different ways depending on communication with the educational centre of origin. Dixon (2014) noted that the interaction and consequences of coordination depend on the staff at the educational centre of origin, giving evidence of success when the relationship is close and communication takes place with a person who is significant for the student. In light of this, the variability seen in our results is striking and might suggest a lack of equity and educational inclusion.

The aim of coordinating with the educational centre of origin, beyond academics, is to keep students in communication with their usual environment, as described by the Hospital Organisation of Pedagogues in Europe (HOPE; 2000) and Benigno and Fante (2020). Maintaining communication and friendship bonds can be of benefit for both students in situations of illness and their classmates (Observatorio Internacional en Pedagogía Hospitalaria, 2022). It can also allow the educational community to become involved and promote learning about illness as part of life (Jiliberto et al., 2022). Indeed, if the goal of educational continuity is to reproduce and compensate for what happens in the educational centre of origin, it is essential to include educational, social and cultural interactions that go far beyond the mere completion of academic work.

It is also necessary for hospital teachers to coordinate with hospital professionals to gather relevant information (Benigno & Fante, 2020; Carstens, 2008; HOPE, 2000; Keehan, 2021). The effectiveness of these interactions influences the quality and individuality of the interventions they deliver (Burns, 2013). Therefore, the disparity in coordination patterns seen in our study is striking, showing that it seems to depend on a given hospital, thereby reflecting variability in each hospital’s sense of the value and need for educational continuity.

Despite HSs being officially recognised educational centres, unlike regular schools, they do not receive annual budgets from the Department of Education. Consequently, and as previously reported by Steinke et al. (2016) and Işiktekiner and Altun (2011), we found a lack of financial provision by education authorities. This lack of annual budgets may limit the effectiveness of the hospital teacher. However, our findings reveal that in some cases this is compensated by the hospital upon request and subjected to approval. Furthermore, all participants reported using personal resources to carry out tasks that otherwise would not be possible, as also observed by Steinke et al. (2016).

Differences were also observed with regard to specific training for hospital teachers. Specifically, 67% of hospital teachers reported not having access to specific training, in line with previous reports (Benigno & Fante, 2020; McNamara, 2024). At the same time, 69% reported receiving supervision and support. Nevertheless, out of these, 22% reported that such services were not quickly accessible or efficient.

Finally, qualitative analysis revealed a lack of coordination between HS in Catalonia, which may seem surprising given the small size of this population and how feasible online meetings for coordination and cooperation purposes would be. Altogether, our results are in line with what Steinke et al. (2016) described as a lack of common structures, organisation, and funding of the different HSs.

### Socioemotional Factors

Results indicate that participants’ perspectives on the competency profile of hospital teachers strongly align with the literature. Teaching in a hospital school setting requires professionals who are willing to cultivate flexibility, serenity, and empathy, as well as being positive and developing the necessary communication skills and emotional stability to be able to manage the diversity and complexity of situations faced in a hospital context. As mentioned by Plage et al. (2022), advocacy should be considered as an additional quality of hospital teachers, as an important safeguard of the right to educational continuity, equity, and inclusion of students in situations of illness.

Our findings regarding the emotional impact of working as a hospital teacher are consistent with those of Benigno and Fante (2020), Keehan (2019), Requena (2015), Bustos (2014), and Carstens (2008), in particular, the bond that is established with both the student and the student’s family. In these circumstances, where life-threatening events occur, the bond established between hospital teacher and student is often intense. Moreover, it can be incorporated into the family and maintained once the illness is over.

Further, our results revealed that hospital teachers perceive situations of deteriorating health and end-of-life processes as highly impactful, corroborating the findings of Keehan (2019), Steinke et al. (2016), Andreatta et al. (2016), and Requena (2015). Similarly, participants considered hospital teaching as a normalising agent for students and recognised the positive consequences this may entail, in agreement with Benigno and Fante (2020), Requena (2015), Burns (2013), and HOPE (2000).

The high satisfaction that participants reported regarding being a hospital teacher is noteworthy, coinciding with the findings of Benigno and Fante (2020), Keehan (2021), Steinke et al. (2016), and Işiktekiner and Altun (2011). Specifically, 92% of the participants consider teaching as a source of personal learning, which corresponds to findings by Bustos and Cornejo (2014) and Carstens (2008).

Our findings also coincide with those of Benigno and Fante (2020), Steinke et al. (2016), and Işiktekiner and Altun (2011) regarding the difficulty in coping with end-of-life situations and yet experience of sense of satisfaction regarding hospital teaching work. It seems that being able to offer a service that helps to alleviate a student’s suffering may be a trigger of satisfaction and personal growth for the hospital teacher. Perhaps, despite having to confront situations of suffering, the hospital teacher values teaching in this context as a way to be in contact with meaningful events that enrich their lifelong learning experience.

### Hospital Context-Related Factors

As mentioned by McNamara (2024), Benigno and Fante (2020), and Ruiz et al. (2020), specific training for hospital teachers is necessary not only in the beginning but on an ongoing basis. For example, with regard to procedures specific to a hospital context, such as infection control protocol, results showed that only 23% of participants received such training upon taking their position.

Further, despite wearing uniforms, hospital teachers are not always easily identifiable. Less than half of the participants considered their uniform easy to recognise and indicative of their role as teachers. The implications of this may extend beyond practical aspects, as students may feel differently towards teachers than towards health professionals.

Not all participants reported feeling a sense of belonging to the hospital. Sixty-nine percent reported feeling included and valued, while 15% expressed not feeling this way. Perhaps the disparity in terms of resources provided by the hospital, coordination with hospital professionals, and supervision and support could explain these results.

### What Happens Next?

Hospital educational care serves as a temporary resource with the goal of maintaining educational continuity and assisting with the student’s re-entry into their original educational centre after convalescence. However, as Barnett et al. (2023) noted, there is uncertainty regarding the efficacy of educational support interventions in enhancing academic performance, school engagement, or the transition back to school. For these reasons, as Ormiston et al. (2024) described, it is necessary to make improvements in the following areas:

- protocols for effective and efficient communication between the educational centre of origin and the hospital teacher,
- reintegration plans addressing academic, social, socioemotional, and medical needs, to be adapted as necessary, and
- enabling the hospital teacher to provide relevant information about the student’s medical condition to prepare the educational centre of origin for facilitating the student’s re-entry.

Altogether, our findings invite us to reflect upon how educational inclusion is managed in this context. Specifically, it may be useful to reflect upon the model that is guiding us, whether it is a medical model based on the individual’s deficit or a social model that acknowledges how social attitudes and barriers imposed by the environment lead to limiting individuals with diverse abilities (Koller and Stoddart, 2021).

Hospitalised students represent a small percentage of the student population attending mainstream schools. Being a minority may pose a challenge in terms of advocacy in itself. However, this is worsened by the fact that hospital students are usually spread out in different schools of origin upon discharge, limiting their chances of gathering and sharing experiences, and, in turn, possibly leading to an inability to recognise common needs or establish advocacy mechanisms.

According to Maslow et al. (2011), the reduction of inequalities, Sustainable Development Goal 10 (UNESCO, 2016), seems not to have been achieved, as young adults who experience chronic illness in childhood have poorer educational, vocational, and financial outcomes compared to their healthy peers – circumstances that can limit their potential of living an independent adult life.

## Conclusion

This study explored the factors that influence education in HSs in an effort to apply this knowledge to improve the experience of hospitalised students. Results revealed that hospital teachers face unique challenges, such as adapting to diverse educational stages and subjects, selecting teaching methods tailored to student needs, and coordinating with various professionals on a case-by-case basis, often without sufficient budgets, consistent access to resources, and professional training. Nevertheless, participants expressed a sense of satisfaction in supporting students while also being affected by emotionally moving situations. Finally, the heterogeneity around factors described as having an impact on education is significant and raise concerns about quality and equity in education. Therefore, it is urgent for policymakers to consider this evidence and reflect upon possible courses of action to amend this.

### Limits of the Study

Despite providing insightful results for an underexplored research area, this study is not without limitations.

First, even though the questionnaire was validated by six field experts, it could be argued that validation from psychometricians, statisticians, and/or survey methodologists was also necessary. However, expert validation was prioritised due to the specialised and situational nature of most questions. Additionally, the questionnaire was strongly tailored to the Catalan context, so its application elsewhere may require adaptations.

Second, although achieving an 87% participation rate relative to the target population, the sample size was too small to estimate correlation coefficients and allow for inferential analysis (Delgado, 2014). However, the focus was placed on descriptive statistics based on the study design, not due to this limitation.

Lastly, when reflecting on the thematic analysis, it is crucial to note that the qualitative data consisted of 2,330 words. Therefore, it is important to recognise that the scale of this analysis was limited compared to more extensive thematic analyses conducted on data from in-depth interviews or focus groups, for example.

### Implications for Further Research

Our findings emphasise the uniqueness of the HS context. Therefore, advancing our comprehension of the factors that influence HS education is crucial for informing decision-making processes among stakeholders and shaping future policies.

To fully understand the HS context beyond the perspective of hospital teachers, future studies should explore the views of other stakeholders. First, of hospital students themselves, since it may not be possible to fully develop an understanding of the factors that influence education in HS without hearing their voices. As Spencer et al. (2023) asserted, the development of policies and the design of interventions should be based on the experiences of the individuals for whom they are intended to be implemented. Additionally, the experience of family members and/or guardians should be investigated, as well as the perspectives of other professionals, such as educational psychologists and social workers.

## Additional Files

The additional files for this article can be found as follows:

10.5334/cie.126.s1 Supplementary File 1. Multi-phase selection process employed in the literature review.

10.5334/cie.126.s2 Supplementary File 2. Emotional impact statements extracted from the literature review.

10.5334/cie.126.s3 Supplementary File 3. Overview of the expert validation process for the questionnaire intended for Catalonian hospital teachers.

10.5334/cie.126.s4 Supplementary File 4. Summary of partial responses.

10.5334/cie.126.s5 Supplementary File 5. Draft English translation of survey questions.
