# Supplementary material for: Influencing Factors In-Hospital School Education: Exploring the Context From the Teacher’s Perspective
Source: Contin Educ. 2025 Jan 31;6(1):1–21. doi: 10.5334/cie.126 (PMC11784520; doi:10.5334/cie.126)
Supplement: Supplementary File 1. — Multi-phase selection process employed in the literature review. [file cie-6-1-126-s1.pdf]

# Influencing Factors in Hospital School Education: Exploring the Context from the Teacher's Perspective

*Supplementary File 1*

**Francisca Jiliberto and Nair Zárate**

**Supplementary File 1. Multi-phase selection process employed in the literature review.**

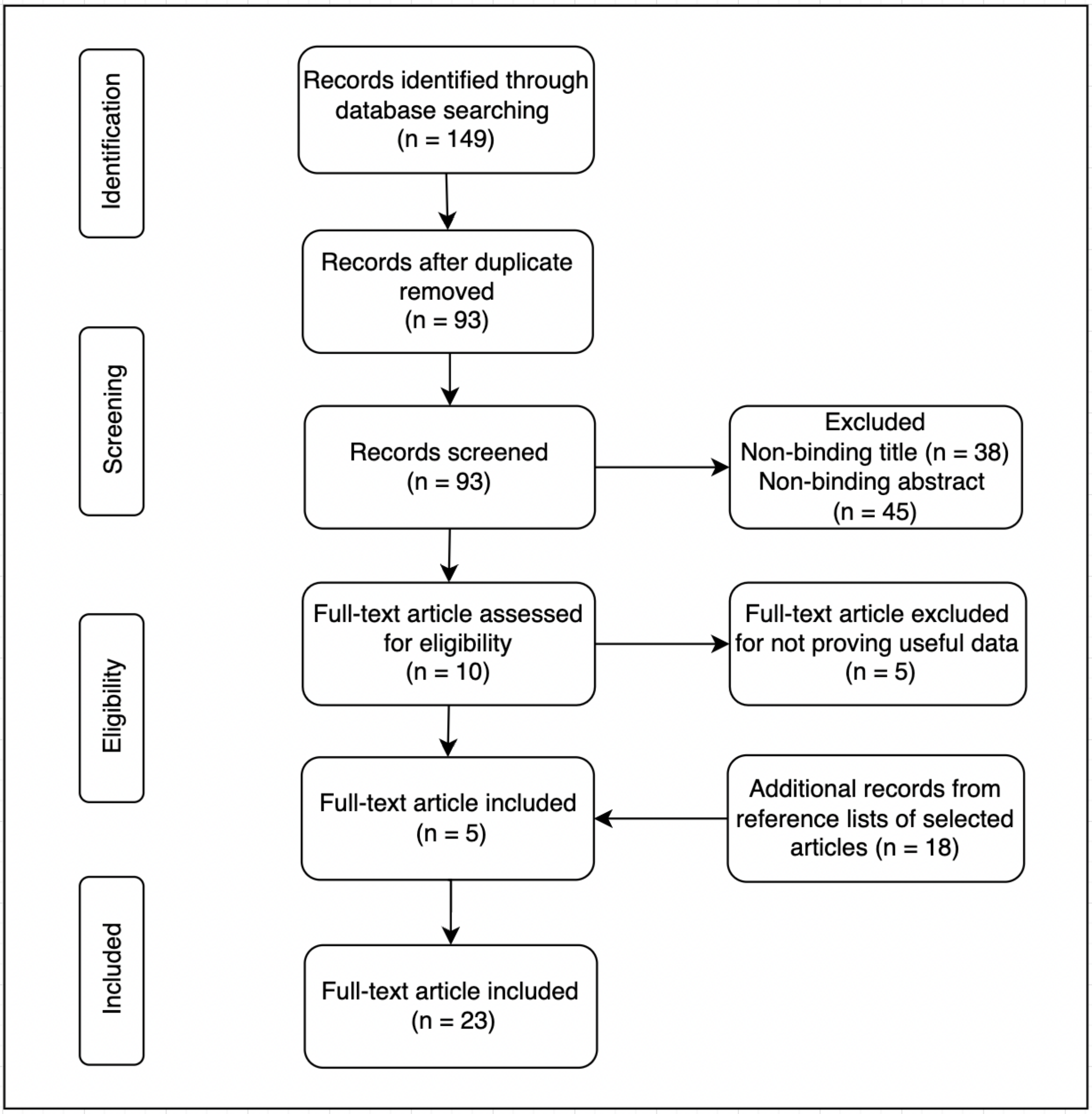


This document contains supplementary material for the above-mentioned article, as provided by the authors.

The original article can be downloaded from https://doi.org/10.5334/cie.126
