# Supplementary material for: Influencing Factors In-Hospital School Education: Exploring the Context From the Teacher’s Perspective
Source: Contin Educ. 2025 Jan 31;6(1):1–21. doi: 10.5334/cie.126 (PMC11784520; doi:10.5334/cie.126)
Supplement: Supplementary File 2. — Emotional impact statements extracted from the literature review. [file cie-6-1-126-s2.pdf]

Influencing Factors in Hospital School Education: Exploring the Context from the Teacher's Perspective

Supplementary File 2

Francisca Jiliberto and Nair Zárate

Supplementary File 2. First-person emotional impact statements related to circumstances hospital teachers may experience as described in the results from the literature review

1. I feel moved when a student's health condition worsens
2. I feel moved when a student goes into palliative care
3. I feel moved when a student starts an end-of-life process
4. I need to share intense emotions; it helps me unload them
5. I often seek emotional support from colleagues or hospital staff
6. I feel satisfaction in regard to being a hospital teacher
7. I feel that working in a HS is a privileged position because of the opportunities it promotes
8. I feel that my role as a hospital teacher helps normalise the exceptional situation that a hospital admission is
9. I feel admiration for the trust and commitment that students demonstrate in spite of their health status
10. I feel satisfaction in being able to accompany students in difficult moments
11. I value the relationships between the hospital teacher and the long-term students; they are usually sincere, intense and affectionate
12. I feel joy when I see that classes can reduce the anxiety, stress and suffering caused by health conditions
13. I share the joy of students and families upon recovery and discharge
14. I am pleased with the gratitude families show for the work I do as a teacher

The original article can be downloaded from https://doi.org/10.5334/cie.126
