# Supplementary material for: Influencing Factors In-Hospital School Education: Exploring the Context From the Teacher’s Perspective
Source: Contin Educ. 2025 Jan 31;6(1):1–21. doi: 10.5334/cie.126 (PMC11784520; doi:10.5334/cie.126)
Supplement: Supplementary File 3. — Overview of the expert validation process for the questionnaire intended for Catalonian hospital teachers. [file cie-6-1-126-s3.pdf]

Influencing Factors in Hospital School Education: Exploring the Context from the Teacher's Perspective

Supplementary File 3

Francisca Jiliberto and Nair Zárate

**Supplementary File 3. Questionnaire validation process**
**First round of validation**
Experts were asked to rate each question from one (low) to four (high) in accordance with the following criteria:<br>a) **Unambiguous**: ensuring that each question had only one interpretation.<br>b) **Relevant**: ensuring that the question allowed for the retrieval of useful information related to the study's aim.<br>c) **Understandable**: ensuring that the question could be clearly understood by the participants.<br><br>Based on the scoring and in accordance with the experts' suggestions 1 item was eliminated, 12 were reframed, and the rest remained unchanged (each multiple-choice question was considered an item, as well as each attribute/statement within Likert-scale question).
**Second round of validation**
Experts were asked to respond to the questionnaire while applying the following criteria:<br>a) Mark with a U if any question was **unclear**.<br>b) Mark with a D if any question was **difficult to answer**.<br>c) Mark with an N if any question is considered **not relevant**.<br>d) Provide **comments** or **suggestions** if necessary.<br><br>No question was rated as unclear, difficult to answer, or not relevant, and comments were positive.

The original article can be downloaded from https://doi.org/10.5334/cie.126
