# Supplementary material for: Influencing Factors In-Hospital School Education: Exploring the Context From the Teacher’s Perspective
Source: Contin Educ. 2025 Jan 31;6(1):1–21. doi: 10.5334/cie.126 (PMC11784520; doi:10.5334/cie.126)
Supplement: Supplementary File 4. — Summary of partial responses. [file cie-6-1-126-s4.pdf]

Influencing Factors in Hospital School Education: Exploring the Context from the Teacher's Perspective

Supplementary File 4

Francisca Jiliberto and Nair Zárate

**Supplementary File 4. Summary of partial responses**
Three items received 11 responses (two participants left it unanswered), and another three items received 12 responses (one participant left it unanswered).
In all cases participants who did not select an option provided a comment instead. Except for two cases:<br>• One participant did not rate the item "harmony with oneself" in the competency profile Liker-scale question.<br>• Another participant did not respond to: "If you have a uniform or distinctive clothing, do you feel that it is easy for students and families to recognise you by your appearance? However, this participant replied not to have a uniform in the previous question.
Percentages for questions with partial responses were calculated based on the number of respondents to that specific question.

The original article can be downloaded from https://doi.org/10.5334/cie.126
