# Supplementary material for: Influencing Factors In-Hospital School Education: Exploring the Context From the Teacher’s Perspective
Source: Contin Educ. 2025 Jan 31;6(1):1–21. doi: 10.5334/cie.126 (PMC11784520; doi:10.5334/cie.126)
Supplement: Supplementary File 5. — Draft English translation of survey questions. [file cie-6-1-126-s5.pdf]

Influencing Factors in Hospital School Education: Exploring the Context from the Teacher's Perspective

Supplementary File 5

Francisca Jiliberto and Nair Zárate

Survey Questions

Please note that the original questionnaire was in Spanish. This translation is provided to facilitate the reader's understanding but has not followed the procedure of a back-translation.

Socio-demographic Questions

1. Age
2. Years of teaching experience
3. Years of teaching experience in hospital schools
4. Hospital school location

Pedagogical Factors

1. In which educational stage do you work?
2. Which subjects do you teach?
3. What pedagogical methodologies do you use during the educational sessions?
4. What type of adaptation do you do the most within the educational sessions?
5. What kind of materials and resources do you use the most during the classes you teach?
6. What do you intend the atmosphere of the class session to be like?
7. How long does a class session usually last per student?
8. After the first contact with the family and the approval of communication with the pupil's school of origin, when does the coordination start?
9. What means of communication do you use to communicate with the pupils' school of origin? Please tick as many alternatives as you think appropriate.
10. How often do you maintain contact with the educational institution of origin in cases of long admissions or long-term treatment?
11. The work plans that I carry out... Please select as many alternatives as you deem appropriate.
12. How many coordination meetings are scheduled in your timetable? Please select as many alternatives as you think appropriate.
13. Do you have financial resources to make available in accordance with professional needs and motivations?
14. Do you have adequate space with the necessary furniture to hold classes?
15. Do you have the necessary technological resources to carry out the educational sessions? Select the alternatives that you think are appropriate.
16. Do you have access to specific and recognised training related to the hospital context?
17. Do you have ongoing supervision and support from the agency that governs your function?
18. How do you consider the professional experience of teaching in a HS?

The original article can be downloaded from https://doi.org/10.5334/cie.126

Socio-emotional Factors: Competency Profile

Mark these attributes as you consider them important.
Very necessary / Fairly necessary / Moderately necessary / Slightly necessary / Not necessary

1. Flexibility
2. Serenity
3. Creativity
4. Dedication
5. Perseverance
6. Patience
7. Happiness
8. Empathy
9. Positivity
10. Communication skills
11. Skills to foster positive personal relations
12. Emotional stability
13. Ability to show kindness.
14. Be open to communication.
15. Be sensitive to the situation of illness.
16. To be in harmony with oneself in order to be able to face the emotional challenges of an HS.
17. Passion for work, students and life.
18. Ability to inspire hope and prospects for the future.
19. Ability to create a positive and harmonious classroom environment.
20. Ability to accept the emotions of the pupil and the family.
21. Ability to generate situations that reduce anxiety levels during educational sessions, creating moments that take away from the situation of illness.

Socio-emotional Factors: Emotional Impact

Check these attributes according to how much you agree.
Totally agree/ Quite agree/ Indifferent/ Quite disagree/ Totally disagree/
Not applicable

1. I feel moved when a student's health condition worsens.
2. I feel moved when a student goes into palliative care.
3. I feel moved when a student starts an end-of-life process.
4. I need to share intense emotions; it helps me unload them.
5. I often seek emotional support from colleagues or hospital staff.
6. I feel satisfaction in regard to being a hospital teacher.
7. I feel that working in a HS is a privileged position because of the opportunities it promotes.
8. I feel that my role as a hospital teacher helps normalise the exceptional situation that a hospital admission is.
9. I feel admiration for the trust and commitment that students demonstrate in spite of their health status.
10. I feel satisfaction in being able to accompany students in difficult moments.
11. I value the relationships between the hospital teacher and the long-term students; they are usually sincere, intense and affectionate.
12. I feel joy when I see that classes can reduce the anxiety, stress and suffering caused by health conditions.
13. I share the joy of students and families upon recovery and discharge.
14. I am pleased with the gratitude families show for the work I do as a teacher.

Hospital Context Related Factors

1.	Do you feel that you are part of the health centre in which the HS is located?
2.	Do you feel that the health centre in which the HS is located spends time and effort in training you on the procedures necessary for the hygiene and safety standards of a hospital?
3.	Do you feel that the health centre in which the HS is located spends time and effort in transferring information relevant to the care of the students?
4.	Do you feel that the health centre in which the HS is located pays attention to your professional needs and are they met?
5.	Do you have adequate working space for non-teaching hours?
6.	Do you have a workspace with the necessary means of communication for the tasks you have to carry out?
7.	Do you have a classroom where group meetings can be held?
8.	Do you make use of personal material resources, when they are not available at your workplace, to meet your work needs?
9.	How do you access information about the number of pupils you have to attend to on a daily basis (list of pupils)?
10.	How many interruptions do you usually have per education session?
11.	Do you feel that your workspace is easy to find within the hospital if someone who does not know you wants to access it?
12.	Do you have a uniform or distinctive clothing provided and maintained by the health facility?
13.	If you have a uniform or distinctive clothing, do you feel that it is easy for students and families to recognise you by your appearance?
